# Effect of Solutionizing Duration and Temperature on the Electrochemical Corrosion and Pitting Resistance of Cold-Rolled Super Austenitic Stainless Steel

**DOI:** 10.3390/ma15248780

**Published:** 2022-12-08

**Authors:** Akeem Yusuf Adesina, Hani M. Ahmed, Rami K. Suleiman, Syed Fida Hassan

**Affiliations:** 1 Interdisciplinary Research Center for Advanced Materials, King Fahd University of Petroleum and Minerals, Dhahran 31261, Saudi Arabia; 2 Materials Science and Engineering Department, King Fahd University of Petroleum and Minerals, Dhahran 31261, Saudi Arabia; 3 Mechanical Engineering Department, King Fahd University of Petroleum and Minerals, Dhahran 31261, Saudi Arabia

**Keywords:** stainless steel, supersaturated austenite, heat treatment, annealing, corrosion, pitting, electrochemical impedance spectroscopy (EIS)

## Abstract

The solution annealing of cold rolled super austenitic stainless steel UN08029 alloy was carried out to investigate the role of solutionizing duration and temperature on the electrochemical corrosion and pitting resistance of the alloy. Linear polarization, cyclic potentiodynamic, and electrochemical impedance spectroscopy techniques were used to evaluate the electrochemical behavior in 3.5% NaCl solution. The microstructural analysis of the solutionized samples revealed the formation of uniform equiaxed grains from elongated columnar grains, which size increases with duration and temperature. The charge transfer resistance shows an increasing corrosion protectiveness of 46 to 60% with increasing solutionizing duration from 30 to 120 min. Similarly, a 45, 52, 60, and 26% improvement in the corrosion performance was obtained for sample solutionized at 1000, 1100, 1200, and 1300 °C, respectively. In general, the solutionized samples demonstrated improved resistance over the as-received alloy, and this behavior increases with solutionizing duration and temperature. Though the pitting potential drops below that of the as-received alloy, the hysteresis loop revealed that the solutionized samples are less prone to pitting damage, and the sample solutionized at 1200 °C for 120 min exhibited optimum pitting corrosion resistance. The microstructural influence on corrosion was also discussed.

## 1. Introduction

Super-Austenitic Stainless Steel (SASS) or High-Alloy Austenitic Stainless Steel (HAASS) is a family grade of stainless steel (SS), mainly with single-phase austenitic microstructure [1]. SASS have attracted immense consideration in several applications in oil & gas, power, petrochemical, and water treatment industries due to their inherently improved mechanical properties (ductility and failure resistance) as well as their superior corrosion resistance in addition to their cost-effectiveness. They are used in highly corrosive environments such as phosphoric acid evaporator tubes, deep and sour gas well production tubing, casing, liners, nuclear power plants, seawater piping, and heat exchangers [2,3]. They are often utilized in applications that require a higher pitting corrosion resistance. In the development of these steels, several alloying elements are added to obtain special and profound properties; however, at certain elevated temperatures, these alloying elements become thermally active and significantly impact on the stabilized phases (austenite and/or ferrite and/or cementite phase) leading to the formation of property-deteriorating intermetallic phases. For instance, the austenitic phase is mainly stabilized when a high percentage of nickel and chromium elements exist [1]. However, at elevated temperatures (usually 550–1050 °C), such as in welding procedures, a range of desirable and undesirable precipitates and secondary phases develop, which can have a significant impact on the mechanical properties and corrosion resistance of the steels [4,5,6,7,8]. The sigma (σ) phase is the most prominent intermetallic phase formed at elevated temperatures [5,6], leading to the decomposition of the austenite through the leaching of chromium and occasionally molybdenum from the material. This results in a deficiency in the uniform distribution of Cr, while the localized formation of Cr-rich compounds, such as carbides, causes the deterioration of the overall corrosion resistance of the steel [5,8].

Yan Jiang et al. [2] investigated the evolution of the sigma phase in a commercial HAASS alloy (N08028), which was water quenched after a 2-h solution annealing treatment at 1200 °C, followed by aging at 900, 950, and 1000 °C for periods ranging from 0.5 to 16 h. They found that the sigma phase initially grows in a granular shape at grain boundaries. At higher periods, it is forced to precipitate with a lamellar structure inside the grain. This sequence occurs due to solute atom redistribution and the difference in thermodynamic driving force and kinetic activation energy coupling effects between inter-granular and intra-granular precipitation. Furthermore, they reported that the volume fraction of the sigma phase increases as the aging temperature decreases and that the distribution position has a significant impact on the morphology of the sigma phase by changing the competition between interface and strain energy [2]. The relationship between microstructure and characteristics of SASS grades S32654 and S31254 was investigated by T. Koutsoukis et al. [5]. Air cooling after heat treatment between 650 and 950 °C, with a step of 100 °C, followed by aging from 0.5 to 3000 h and then water quenched. After the aging period, the study revealed four different types of precipitates: σ-phase, χ-phase, Laves phase, and β-Cr2N nitride. Results showed that Laves phase is the first secondary phase to form, followed by the σ-phase, then the χ-phase, and lastly, β-Cr2N (which requires a higher aging time to form). At an aging temperature of 950 °C, the full change of the Laves phase to the sigma phase and/or dissolving in the matrix happen within 240-h. In both alloys, precipitation of predominantly Laves phase and σ-phase resulted in an increase in hardness values at all temperatures [5]. The influence of heat treatment on the microstructure and corrosion behavior was investigated by L.N. Zhang et al. [4]. For the same aging time, they reported that the precipitation rate is higher at 900 °C than at 850 and 950 °C. According to the thermodynamic calculations presented in [4], the σ-phase begins to form at about 400 °C and reaches its maximum at a temperature around 600 °C, and completely dissolves at a temperature higher than 1100 °C. Though carbides also initially form at similar temperatures, they remain quite low throughout the precipitation temperature range (450–1100 °C). Hence, the sigma phase is the most important component in precipitation in SASS. Furthermore, as aging time increases, the intermetallic precipitation and, thus, the hardness also increases. Similarly, when the amount of precipitates increases, the corrosion rate increases, implying that the presence of precipitates reduces corrosion resistance [4]. Anburaj et al. [9] reported the behavior of Alloy-7Mo heat treated at 1250 °C for 3 h and then aged at 500 to 1000°C for 1 h and 10 h. At 900°C, sigma is richer in Mo and can be deleterious to SASS toughness due to susceptibility to de-cohesion with the austenite matrix [9]. The influence of cooling rate following heat treatment on the pitting corrosion of UNS S32750 was investigated by Byung-Hyun Shin et al. [10]. The austenite volume fraction increased while the ferrite volume fraction fell when the cooling rate was reduced from 5600.0 J/s (water cooling) to 1.9 J/s (air cooling) at the same temperature. At all temperatures, lowering the cooling rate to 0.4 J/s (furnace cooling) improved the precipitation of the secondary phases [10].

Therefore, it is evident that some studies [5,6,7,8,9,10] have been conducted which investigate the effect of aging on the microstructure and corrosion properties of super austenitic stainless steel. However, to the best of our knowledge, no study has been conducted to investigate the effect of duration and temperature during solution annealing (solutionizing) on the corrosion properties of SASS UNS N08029 grade. Thus, this study aimed to investigate the influence of solutionizing duration and temperature during heat treatment on the electrochemical behavior and pitting resistance of the alloy UNS N08029 in a 3.5% NaCl corrosive environment.

## 2. Materials and Methods

### 2.1. Sample Preparation and Solutionizing Treatment

**Figure 1:**
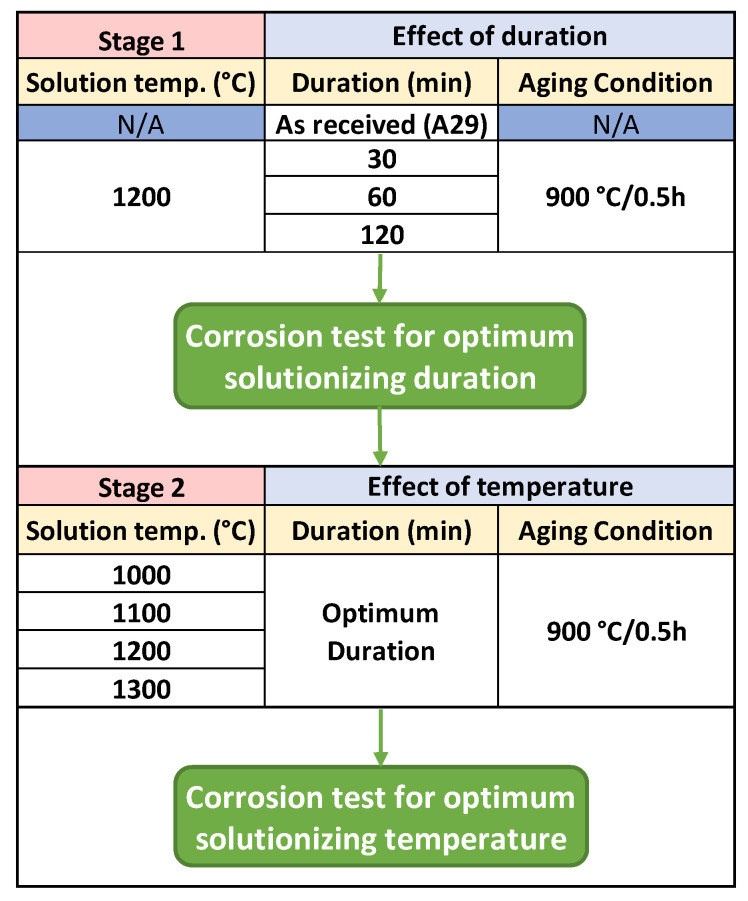
Experimental matrix and flow chart showing the sequence of the heat treatment and corrosion testing.

Super austenitic stainless steel (SASS) samples with nominal composition, as shown in Table 1, were obtained in the cold-hardened condition. The samples were then solution annealed after sectioning to appropriate dimensions. A tube furnace (GSl-1700X, MTI, Richmond, CA, USA) was utilized for conducting the solutionizing heat treatment. In the first stage of the heat treatment, the samples were solutionized at 1200 °C for different durations of 30, 60, and 120 min and tagged as T30, T60, and T120, respectively. This treatment stage aimed to assess the effect of solutionizing duration on corrosion behavior. Thereafter, an optimum duration based on the corrosion evaluation was maintained for the second stage of the solutionizing heat treatment. This second stage targeted understanding the influence of solutionizing temperature on the corrosion behavior of SASS N08029 alloy. The samples were then solutionized at different temperatures of 1000, 1100, 1200, and 1300 °C for 120 min and then tagged as AT10, AT11, AT12, and AT13, respectively. The detailed solution annealing heat treatment program showing the relation between the two stages is schematized in Figure 1. All solution-annealed heat-treated samples were quenched in water to room temperature and thereafter aged at 900 °C for 0.5 h before they were air cooled to room temperature and submitted for further characterization and testing.

### 2.2. Characterization of As-Received and Solutionized Samples

All solutionized samples were subjected to grinding using SiC papers with sizes ranging from 240 to 800 grit size to remove all oxides layer resulting from the heat treatment. Thereafter, the samples were polished to mirror-like surfaces for characterization and corrosion testing. To reveal the microstructure, the samples were electrolytically etched using electrolytic polishing and etching instrument (Electro-P, Echo Lab, DEVCO srl, Paderno Dugnano, MI, Italy). The samples were etched in equal proportions of HNO_3_ and distilled water at a current of 0.6 A at a voltage of 12 V for 30 s under ambient conditions. Prior to the electrolytic etching, the samples were ground on silica paper of different grit sizes and polished to a mirror finish using 0.1 µm diamond paste on emery cloth. The optical images were obtained using an optical microscope (Video, Microcombi, CMS Instruments, Switzerland), while ImageJ image analysis software (v. 2.9.0) [11] was used to conduct the grain size analysis. To investigate the structural evolution, an X-ray diffractometer (XRD, Rigaku MiniFlex, Tokyo, Japan) was used with Cu Kα1 radiation (γ = 0.15416 nm), a tube current of 10 mA, and an accelerating voltage of 30 kV. The microstructural evolution resulting from the different heat treatment conditions was investigated using an emission scanning electron microscope (SEM, JEOL, Tokyo, Japan) with an accelerating voltage of up to 20 kV coupled with energy-dispersive X-ray spectroscopy (EDX) silicon drift detector (X-MaxN, Oxford Instruments, Abingdon, UK).

### 2.3. Electrochemical Corrosion Measurements

Electrochemical corrosion measurements were conducted to reveal the corrosion behavior and the effect of the adopted heat treatment conditions. A three-electrode system was utilized for the corrosion test wherein the samples served as the working electrode, an Ag/AgCl electrode was used as the reference electrode, and a graphite rod was utilized as the counter electrode. For the evaluation of the optimum solutionizing duration and temperature, the corrosion behavior of all samples was studied in chloride environments by employing 3.5% (0.6 M) NaCl aerated solution under ambient conditions using a flat sample with a dimension of 30 × 30 × 5 mm. The exposed area for corrosion study is 2.85 cm^2^, and an immersion duration of 1 h for the system to attain steady-state condition during which the open circuit potential (OCP) was measured before the commencement of other electrochemical tests. The electrochemical measurements were conducted using a Gamry potentiostat (Model 600, Gamry, Warminster, PA, USA). Potentiostatic electrochemical impedance spectroscopy (EIS) measurement was carried out by applying a sinusoidal excitation voltage of ±10 mV and acquiring the impedance responses over a frequency range from 10^5^ to 10^−1^ Hz. Similarly, the linear polarization resistance (LPR) measurement was conducted over a voltage range of ±25 mV against the OCP with a scan rate of 0.125 mV/s. The cyclic potential dynamic polarization (CPDP) measurement was carried out as per ASTM standard guidelines [12]. The CPDP test involves perturbing the system from an initial potential of −0.5 V against the OCP with a forward scan rate of 0.5 mV/s until an apex current or voltage of 5 mA/cm^2^ or 1.5 V, respectively, whichever is first reached. Then reverse scanning is conducted at a scan rate of 0.5 mV/s until a final potential of 0.5 V against the OCP is attained. Each corrosion measurement was conducted at least twice to ensure repeatability.

## 3. Results

### 3.1. Characterization of the Solutionized Samples

**Figure 2:**
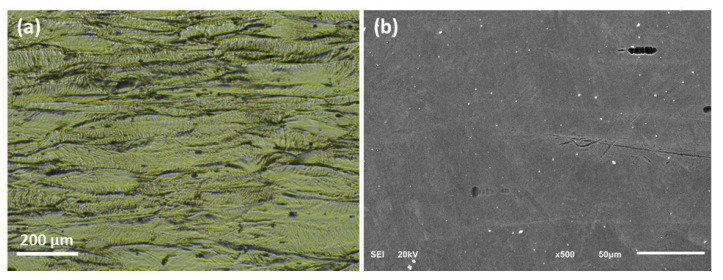
(**a**) Optical and (**b**) SEM images of the as-received cold rolled UN08029 alloy showing the deformed columnar grains.

The optical micrograph and the SEM images of the as-received UN08029 alloy (sample A29) showing the deformed columnar grains resulting from the cold-rolled process are depicted in Figure 2. The horizontal direction is the rolling direction, and it is evident that the sample has undergone extensive deformation from rolling as the interface between the deformed grains is almost invisible; thus, the etching of the sample becomes very challenging.

**Figure 3:**
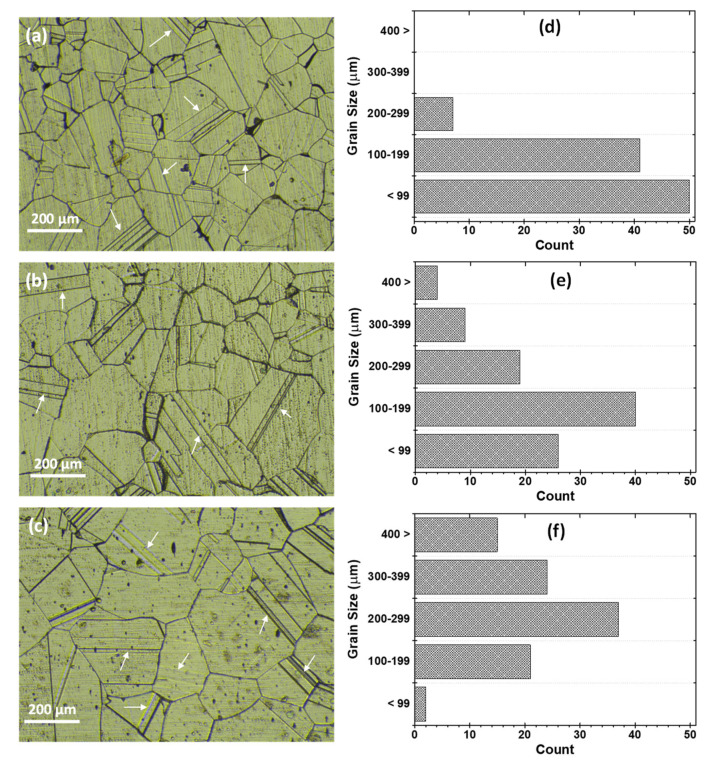
(**a**–**c**) Optical micrographs and (**d**–**f**) grain size distribution for heat-treated samples (**a**,**d**) T30, (**b**,**e**) T60, and (**c**,**f**) T120.

While in Figure 3, the optical images and the grain size distribution are presented, revealing the effect of the different solutionizing durations on the microstructure. After solution heat treatment at 1200 °C for different durations (30, 60, and 120 min), crystallization to equiaxed grains with different sizes was observed from the deformed and elongated grains of the as-received alloy (Figure 3a–c). Analysis of several optical micrographs indicates that there is grain growth that accompanies the increase in solution duration. Figure 3d–f shows the grain size distribution as a function of solutionizing duration. It is observed that the majority of the grains of sample T30 (solutionized for 30 min) are below 99 µm, about 40% are between 100 and 199 µm, and the remaining 10% are between 200 and 299 µm. For the sample T60 (solutionized for 60 min), the grain size distribution shows that about 25% of the grains are below 99 µm, about 20% are between 100 and 299 µm, and 15% are above 300 µm while for the sample T120 (solutionized for 120 min), it shows that less than 2% are below 99 µm, about 40% are between 200 and 299 µm and 38% are above 300 µm. This results in an average grain size of 108, 177, and 280 µm as the solution duration increases from 30, 60, and 120 min, respectively. The grain growth with solutionizing duration can be associated with particle coalescence due to increased thermal energy by diminishing the grain boundary area per unit volume [13,14].

**Figure 4:**
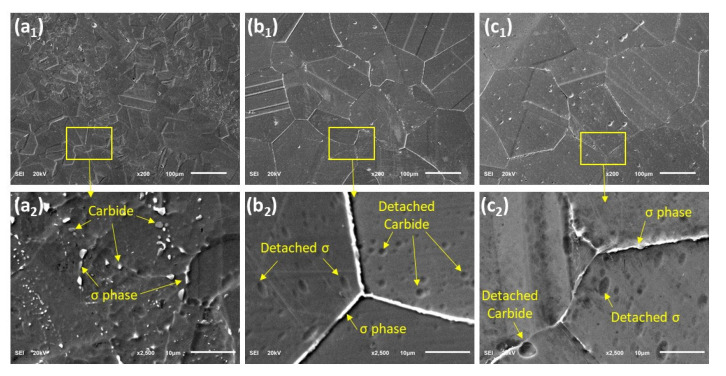
SEM images showing the grains and the grain boundaries for heat-treated samples (**a_1_**,**a_2_**) T30, (**b_1_**,**b_2_**) T60, and (**c_1_**,**c_2_**) T120.

Figure 4(a_1_–c_1_) shows the SEM images, which also substantiate the increasing grain size as the solution duration increases. Similarly, the higher magnification SEM images revealed the presence of precipitates, as apparent in Figure 4(a_2_–c_2_). EDS analysis of these precipitates showed that they are mainly carbides and sigma (σ) phases (Table 2). The σ phase is more of a needle-like shape, while the carbide is more spherical. For the sample solutionized for 30 min (T30), the precipitates are relatively smaller and randomly distributed within the grains. However, for the samples (T60 and T120), the precipitates are found mostly on the grain boundaries, with no visible precipitate inside the grains. Because it contains high concentrations of chromium and molybdenum, the sigma phase appears in a lighter shade of gray [15]. Furthermore, twin boundaries can be observed from the optical micrographs (shown with arrows in Figure 3). The twin boundaries are a special case of a large angle grain boundary whereby there is no misalignment of atoms, and thus adjacent planes are mirrored across the boundary. The twin boundaries are found to increase with solution inning duration, as evident from the optical images as well as from the SEM images in Figure 4.

**Figure 5:**
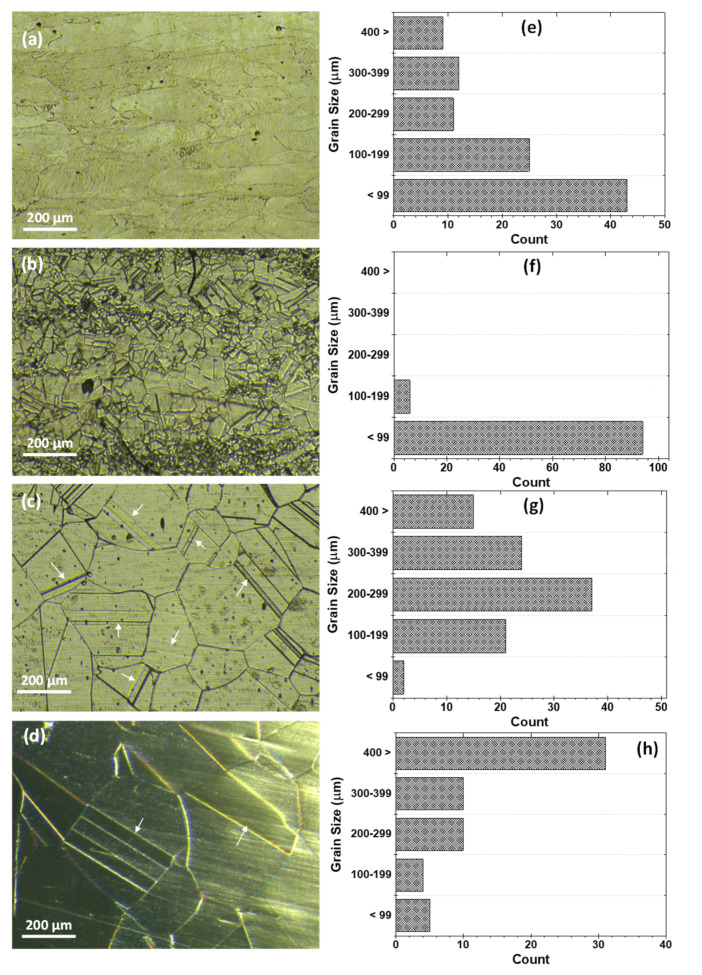
(**a**–**d**) Optical micrographs and (**e**–**h**) grain size distribution for heat-treated samples (**a**,**e**) AT10, (**b**,**f**) AT11, (**c**,**g**) AT12, and (**d**,**h**) AT13.

Figure 5 shows the optical images, as well as the grain size distribution of the samples, solutionized at different temperatures. The effect of the temperature on the microstructure is evident in the images. The sample solutionized at 1000 °C (Figure 5a) is similar but with well-defined grain boundaries as opposed to the microstructure of the as-received sample (shown in Figure 2). This is the initial stage in the formation of the equiaxed microstructure. Further increase in the solutionizing temperature to 1100 °C revealed a complete transformation of the cold rolled columnar grains to equiaxed microstructure with fine grains, as shown in Figure 5b. The grain size increases with a further increase in the solutionizing temperature, as seen in the micrographs in Figure 5a–d. At a constant dwell duration of 120 min, an increase in the solutionizing temperature will induce an increase in the thermodynamic driving force and kinetic activation energy, which accelerate grain coalescence and growth [2]. Figure 5e–h shows the grain size distribution for the samples solutionized at different temperatures. The grains of sample AT10 (Figure 5e) are mostly still elongated columnar grains, and thus, an average of the length and breadth of each elongated grain was considered in this analysis. Furthermore, it should be noted that the comparison with the other solutionized samples cannot be directly made as the sample has not been recrystallized into equiaxed grains. However, the samples solutionized at temperatures of 1100, 1200, and 1300 °C have been fully recrystallized, and their grain size can be compared accordingly. Thus, it is observed that the majority of the grains of sample AT11 (solutionized at 1100 °C) are almost below 99 µm, while the sample AT12 (solutionized at 1200 °C), the grain size distribution shows almost a uniform distribution with about 25% of the grains are below 200 µm, about 40% are greater than 300 µm, and 38% are between 200 and 299 µm. Sample AT13 (solutionized at 1300 °C) shows that about 35% of the grains are above 400 µm, about 30% are between 200 and 399 µm, and 10% are below 199 µm. Consequently, the average grain size of samples solutionized at temperatures of 1100, 1200, and 1300 °C was found to be 42, 280, and 392 µm, respectively. Elevated solutionizing temperatures accelerate grain boundary migration due to increased thermal energy leading to grain growth [13,14]. It was seen that recrystallization and grain growth were the main mechanism as the solutionizing temperature increased. Furthermore, after a detailed observation of the optical images, it is interesting to note that the sample solutionized at 1200 °C possessed the largest number of twin boundaries compared withall the solutionized samples, as seen in Figure 5. Twin boundaries are known for their very low energy due to their high degree of order at the interface, providing some superior properties compared with random high-angle grain boundaries [16].

**Figure 6:**
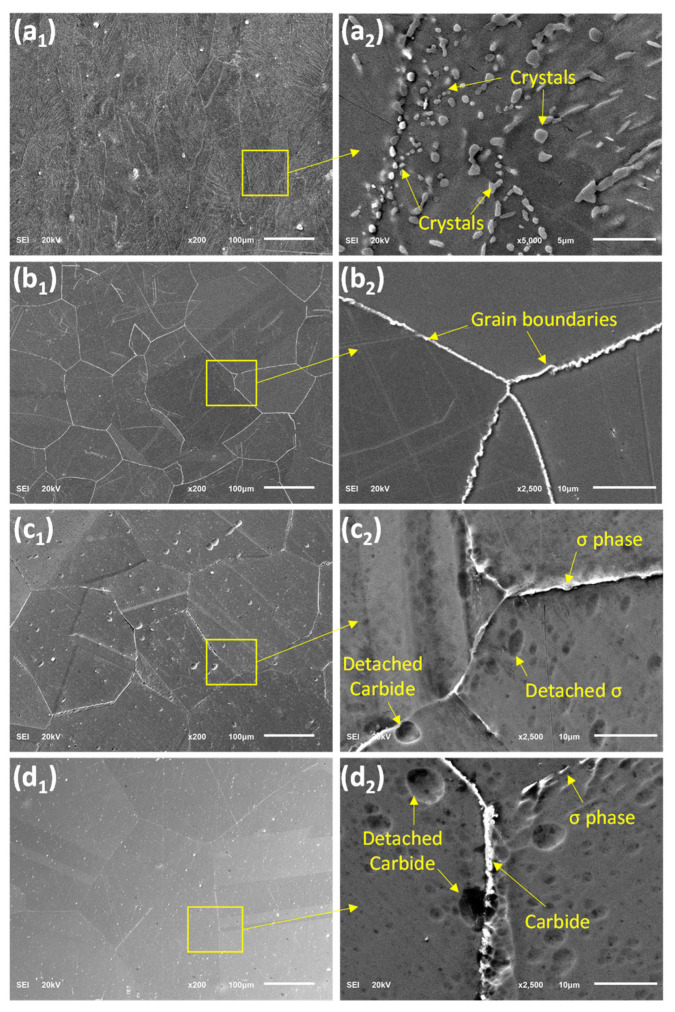
SEM images showing the grains and the grain boundaries for heat-treated samples (**a_1_**,**a_2_**) AT10, (**b_1_,b_2_**) AT11, (**c_1_**,**c_2_**) AT12, and (**d_1_**,**d_2_**) AT13.

The SEM images of samples solutionized at different temperatures are shown in Figure 6. A closer look into the SEM images of sample AT10 in Figure 6(a_1_) shows numerous particle-like features within the elongated columnar grains, which can be identified as sub-grains or fine crystals; these are shown with the high magnification SEM images in Figure 6(a_2_). These fine crystals are usually formed mostly during the high-temperature recovery process [17]. With the increment of solutionizing temperature, the crystals coalesce to form larger grains. At a higher solutionizing temperature of 1200 °C, it is apparent that the crystals have become fully equiaxed, as shown in Figure 6(b_1_,b_2_), and continue to grow with increasing temperature, as evident in Figure 6(c_1_,d_1_). It is interesting that at higher solutionizing temperatures, secondary phases precipitates of sigma and carbide phases can be observed in Figure 6(c_2_,d_2_), which seems to be more at a higher temperature. It has been observed that the higher the annealing temperature, the faster the grain boundary mobility, the sooner the grain growth occurs, and the larger the grain size [17].

### 3.2. Electrochemical Corrosion Behavior of Solutionized Alloy 29

#### 3.2.1. Open Circuit Potential (OCP) Measurement

**Figure 7:**
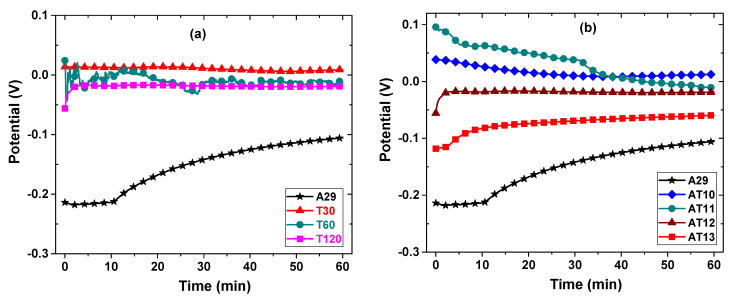
(**a**) Open circuit potentials showing the effect of (**a**) solutionizing duration and (**b**) solutionizing temperature on the corrosion behavior of N08029 after 1 h immersion in 3.5% NaCl aerated solution.

The OCP measurement was used to monitor the unperturbed corroding potential of the sample when no external current was applied, and the system is expected to attain a steady-state condition when the potential is no longer a function of the immersion duration. Thus, the OCP shows the stability of the electrochemical environment before polarizing the system, and it also provides information about the thermodynamic tendency of the samples toward corrosion. The OCP of the solutionized samples is shown in Figure 7, and it is evident that the samples attained an equilibrium state after the monitoring duration of 1 h. Figure 7a shows the OCP trend of the samples solutionized for different durations (30 to 120 min). It is seen that the samples attain an equilibrium state and exhibit a nobler potential in comparison to the untreated alloy (A29). Furthermore, the potential of the sample T30 is noblest, and as the solutionized duration increase from 60 to 120 min, the potential reduces slightly from −11.4 to −18.8 mV compared with a potential of −105.6 mV of the as-received alloy (Sample A29) at the end of 1 h monitoring duration. A similar trend is observed for the samples solutionized at different temperatures (1000 to 1300 °C), as shown in Figure 7b. Sample solutionized at 1000 °C (sample AT10) shows the noblest potential and increasingly becomes active (less noble) with solutionizing temperatures. The most active sample (sample AT13) showed a potential of −59.1 mV compared with sample A29, which exhibited a stable potential of −105.6 mV at the end of the 1 h monitoring duration. Besides, the trend in the OCP can also be related to the formation of the passive film. The lower and increasing OCP with time for the A29 sample indicates the incremental film formation over time. However, the solutionized samples mostly showed a steady OCP over a wide duration, indicating the early formation of the film and its stability and protectiveness. Generally, the degree of the nobility of the OCP after attaining equilibrium indicates the inertness of the sample, and hence the lower the corrosion propensity in the test environment.

#### 3.2.2. Linear Polarization Measurement

**Figure 8:**
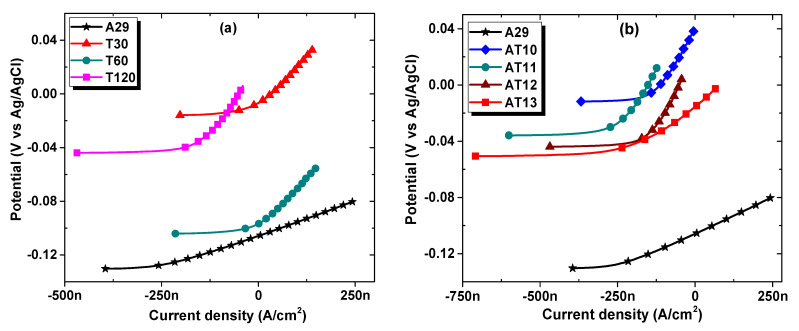
LPR curves showing the effect of (**a**) solutionizing duration and (**b**) solutionizing temperature on the corrosion behavior of UN08029 after 1 h immersion in 3.5% NaCl aerated solution.

Figure 8 shows linear polarization resistance (LPR) plots for the as-received and solutionized samples. From the LPR curves, it can be observed that all the solutionized samples possessed higher corrosion resistance than the as-received cold-rolled UN08029 alloy, as evident from the steepness of the slope of the potential vs. current curves. The corrosion resistance is directly proportional to the slope of the LPR curve. It is often used to estimate the corrosion current density (*I_corr_*) according to the Stern Geary relationship as in Equation (1) [18,19]. Accordingly, the polarization resistance (R_p_) was obtained from the slope of the LPR curves, while the *I_corr_* and corrosion rate (*CR*) were then obtained from Equations (1) and (2), respectively [18,19].

(1) $$I_{corr}=\frac{\beta_{a}\beta_{c}}{2.303{\;R}_{P}\left(\beta_{a}+\beta_{c}\right)}$$

where R_p_ is the polarization resistance obtained from the LPR slope, and βc and βa are the cathodic and anodic Tafel constants taken as ±0.12, respectively.

(2) $$CR=\frac{\;0{.131*\;I}_{corr}*EW}{\rho }$$

where ρ is the density, and *EW* is the equivalent weight of the UN08029 alloy.

The parameters R_p_ and I_corr_ for the samples obtained from the LPR measurement are shown in Table 3 and Table 4 showing the effects of solutionizing duration and temperatures, respectively. All the solutionized samples demonstrated a higher slope, thus larger R_p_ and consequently lower I_corr_ than the as-received UN08029 alloy (A29). As seen from Table 3, as the solutionizing duration increases from 30 to 120 min, the R_p_ increases. This necessitates a corresponding reduction in the current density and corrosion rate with an increase in the solutionizing duration. The R_p_ of the untreated alloy sample was increased by about 67, 68, and 76% after solutionizing for a duration of 30, 60, and 120 min, respectively, as estimated from the resistive efficiency (ε_lpr_) according to the LPR measurement. Though there is an appreciable increase in the Rp compared with the untreated sample A29, the increment among the solutionized samples is not that significant, especially for the duration between 30 and 60 min, as the grain size distribution is fairly similar (see Figure 3). The increasing corrosion resistance behavior can be associated with the microstructure of the samples after solutionizing for the different duration, as shown in Figure 3 and Figure 4. An increase in the grain size with solutionizing duration reduces the grain boundary defects and thus improves the corrosion resistance. Though there are precipitated carbide and sigma phases, as observed in Figure 4(a_1_,a_2_), this does not seem to affect the corrosion behavior of this alloy. This may be attributed to the substantial availability of Cr, Mo, and Ni in the matrix (as seen in Table 2), which are instrumental in the corrosion resistance property, so the matrix was not detrimentally depleted of these elements for the formation of the secondary phases.

The LPR parameters for the sample solutionized at different temperatures are shown in Table 4, and the I_corr_ is lower for the solutionized samples, with sample AT12 exhibiting the lowest values. In addition, it is seen that the R_p_ of the untreated alloy sample (A29) was increased by about 72, 74, 76 and 42% after solutionizing at temperatures of 1000, 1100, 1200, and 1300 °C, respectively, as enumerated from the ε_lpr_. The obtained resistive efficiency of 72% for sample AT10 increases slightly until 1200 ^o^C and then drops to about 42% at 1300 °C (sample AT13). Similarly, the increased corrosion resistance with increasing solutionizing temperature can be linked to grain size growth, as evident from Figure 5. However, the reduction in the corrosion behavior at a temperature of 1300 °C may be attributed to the increased growth and coalesces of secondary phases in the matrix as well as wider grain boundaries of carbides and sigma phases, as shown in Figure 6. The increased secondary phases are associated with the lower number of twin boundaries than that of sample AT12. Since twin boundaries are highly resistant to carbide precipitation during treatments [16], it is expected that a sample with a lower number of twin boundary samples will exhibit a percentage of carbide precipitates.

#### 3.2.3. Cyclic Potentiodynamic Polarization Measurement

**Figure 9:**
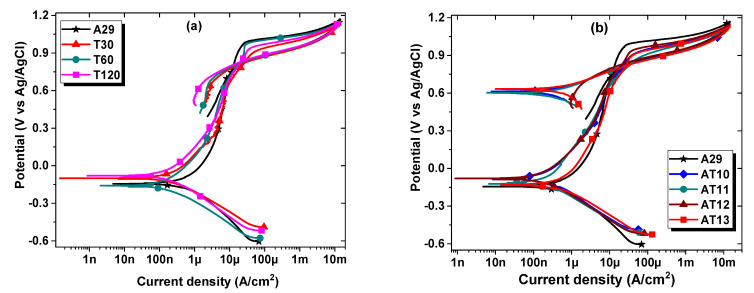
CPDP curves showing the effect (**a**) solutionizing duration and (**b**) solutionizing temperature on the corrosion behavior of UN08029 after 1 h immersion in 3.5% NaCl aerated solution.

The CPDP measurement also revealed important corrosion characteristics of the as-received and solutionized samples. The Tafel extrapolation method was employed to obtain the corrosion potential (E_corr_) and corrosion current density (I_corr_) from which the corrosion rate (CR) was calculated. The CPDP plots for the solutionized samples are shown in Figure 9, and they exhibited both active and passive behavior at various polarizing potentials. The parameters E_corr_, I_corr_, and CR quantify the general corrosion behavior of the samples, while the pitting potential (E_pit_), passivation current density (I_p_), and the repassivation or protection potential (E_p_) are essential parameters for quantifying the localized pitting corrosion behavior of the samples [12,20]. The E_pit_ is defined as the potential at which the surface passive film breakdown occurs, as indicated by the rapid increase in the current in the active-passive region [12,20,21]. For a better insight into the film breakdown, the Ip was determined as the corresponding current density at which the surface film breakdown occurs. While the E_p_ is defined as the potential in the reverse scan direction when repassivation occurs or the hysteresis loop (approximated to E_pit_ – E_p_) is completed (i.e., when the reverse scan intercepts the forward scan) [20].

Figure 9a is the CPDP plot for the samples solutionized for different time durations, and the samples exhibited similar behavior. However, all the solutionized samples were shifted to lower current density while the E_corr_ of the samples T30 and T120 became nobler than the as-received sample. Subsequently, the solutionized samples showed higher potential at any given current density almost throughout the passive region. Though the E_pit_ for the untreated sample A29 is slightly higher than for the solutionized samples, a closer look into the hysteresis loop shows that the loop size is in the decreasing order of T30 < T120 < T60 < A29. The lower the hysteresis loop, the faster the repassivation and the lesser the damage due to pitting corrosion on the sample. Table 5 shows the quantitative CPDP parameters for the solutionized samples for a different duration. It is evident from the table that the I_corr_ and, consequently, CR decreases, necessitating an increase in resistance efficiency (ε_cp_) with solutionizing duration and consonant with the LPR findings. The E_pit_, I_p_, and E_p_ values which quantify the pitting corrosion behavior, show that the as-received sample A29 exhibited the highest E_pit_ and lowest I_p_. However, it has the lowest E_p_ values. This indicates that though the pitting potential is the highest and occurs at a lower current density, repassivation is somewhat delayed, which means more pitting corrosion damage to the sample. Based on the repassivation capability, samples T30 and T120 possessed improved repassivation tendencies which resulted in lesser pitting damage, especially considering that their I_p_ values are just barely higher than that of the as-received sample.

Similarly, the CPDP plots for the samples solutionized at different temperatures are shown in Figure 9b, and the E_corr_ of the solutionized samples shifted to the noble positive potentials with correspondingly lower I_corr_. This behavior shows that all the samples solutionized at different temperatures exhibited improved resistance against general corrosion in comparison with the as-received sample A29. Furthermore, for the pitting behavior, the as-received sample offered the highest E_pit_ and lowest I_p,_ just as observed with the samples solutionized for a different duration. However, the hysteresis loop of sample A29 is the largest due to its E_p_ value being the least. Interestingly, the hysteresis loop for the solutionized samples is almost overlapping, and the E_p_ is only slightly different. The E_corr_, I_corr_, and ε_cp_ parameters, alongside the pitting corrosion parameters for these samples, are given in Table 6. The ε_cp_ parameter can be seen to be within a close range between 84 and 87% with the solutionizing temperature and then sharply drops to 55% for the sample solutionized at 1300 °C (sample AT13). The samples can be ranked according to increasing E_pit_, thus, A29 > AT12 > AT13 > AT10 > AT11, while the hysteresis loop can be ranked in the increasing order thus: A29 > AT12 > AT13 > AT10 > AT11. It should be noted that the sample with a high E_pit_ and a low hysteresis loop will demonstrate superior pitting resistance. Thus, it can be proposed that sample AT12 showed an optimum combination of both E_pit_ and a relatively low hysteresis loop, therefore will suffer less from pitting corrosion damage.

#### 3.2.4. Electrochemical Impedance Spectroscopy Measurements

Electrochemical impedance spectroscopy (EIS) is probably among the most sophisticated non-destructive steady-state electrochemistry techniques used for corrosion evaluation as it can provide information relating to the reaction parameters, corrosion rates, oxide formation, and their characteristics, surface integrity, and porosity as well as other interfacial properties [22,23]. Thus, the investigation of the corrosion behavior of the as-received and heat-treated alloys using EIS was also conducted.

**Figure 10:**
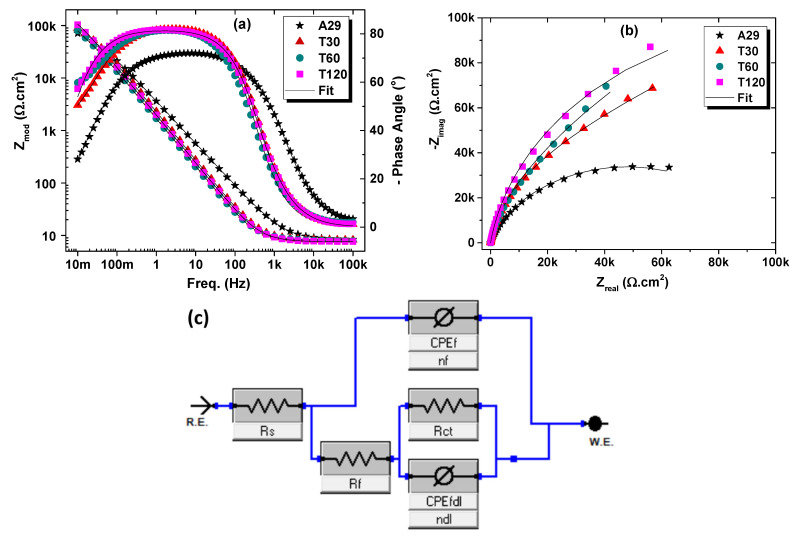
EIS plots, (**a**) Modulus and phase angle, (**b**) Nyquist plots, showing the effect of solutionizing duration on the corrosion behavior of N08029 after 1 h immersion in 3.5% NaCl aerated solution, and (**c**) Equivalent circuit used in fitting the EIS data.

**Figure 11:**
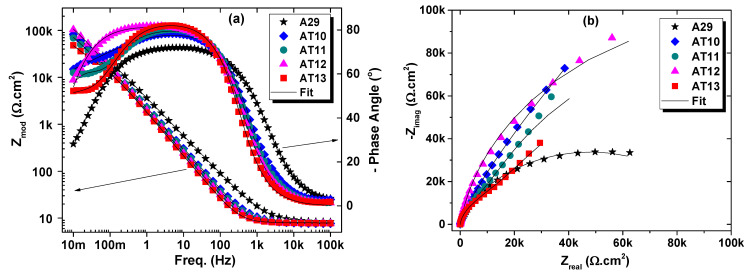
EIS plots, (**a**) Modulus and phase angle, and (**b**) Nyquist plots, showing the effect of solutionizing temperature on the corrosion behavior of A29 after 1 h immersion in 3.5% NaCl aerated solution.

Figure 10 and Figure 11 are the Bode and Nyquist EIS plots showing the effect of solutionizing duration and temperature on the electrochemical behavior of the alloy, respectively. It can be observed from the Bode plots (which consist of modulus impedance (|Z|) and phase angle (θ) plots) that the log |Z| vs. log f (frequency) relationship does not conform to −1. The phase angle maxima (θ_max_) is much below −90 degrees, indicating a deviation from ideal capacitive behavior. It is known that the |Z| measured from the low-frequency portion of the modulus impedance plot is related inversely to the corrosion rate; thus, a larger absolute impedance assures superior charge transfer resistance at the surface/electrolyte interface [24,25,26,27].

In Figure 10a, the |Z| can be observed to increase with increasing solutionizing duration. At the same time, the θ_max_ also tends to increase fairly above −80 degrees in comparison with the as-received alloy, which is just about −70 degrees. The higher the θ_max,_ the higher the capacitive behavior of the alloy, which indicates improved resistance to charge leakage or transfer to the corrosive medium. Similarly, the peak of the θ_max_ for the solutionized samples is also observed to shift to a lower frequency than that of the as-received alloy. This may be associated with surface film formation resulting from corrosion. The accompanying broadening of the phase angle plot with solutionizing duration indicates a two-time constant phenomenon [22]. Figure 10b shows the Nyquist plot, which supports the increment with solutionizing duration evident from the increasing diameter of the incomplete semi-circle capacitive arc. The higher the diameter of the semi-circle, the higher the total resistance to corrosion which indicates the protective performance of the alloys in the medium under consideration [26,27].

Similarly, the samples solutionized at different temperatures demonstrated an increasing |Z|, a higher θ_max_, and a broader phase angle plot with temperature until 1200 °C (sample AT12), and these parameters then decrease at the solutionizing temperature of 1300 °C (sample AT13) as shown in Figure 11a. This is also seen in the Nyquist plot in Figure 11b from the increasing diameter of the incomplete semi-circle capacitive arc with temperature until 1200 °C after which the capacitive arc reduces. It is interesting to note that the θ_max_ of sample AT13 does not drop. Rather the broadening of the phase angle only reduces. This illustrates the mechanism and reason for the observed reduction in the protectiveness of this sample, and this can be associated with the oxide film instability and not being compact or dense enough in comparison to the other solutionized samples.

The EIS data were fitted with an equivalent circuit (EC) to enumerate the various parameters useful in further understanding the electrochemical behavior of the solutionized samples. An observation of the EIS plots shows that the samples exhibited a two-time constant phenomenon as seen from the two inflection points and/or wideness of the phase angle plots of the Bode diagram. This will be more evident if allowed for more time or at a lower frequency. Thus, the EC consisted of a constant phase element accounting for the non-ideal capacitive behavior of the electrical double-layer (CPE_dl_). The CPE_dl_ is connected in parallel with the surface resistance to charge transfer (R_ct_). Both CPE_dl_ and R_ct_ are further connected serially to the resistance of the oxide films (Rf) and a constant phase element (CPE_f_), which accounts for the capacitive properties of the oxide film. The solution resistance (Rs) is connected in series with the R_f_ and CPE_f_ elements. The EC used in this study is shown in the insert of Figure 10c as well described as Model C in [28], and it is from the most famous EC used for fitting EIS data of stainless steels [29,30,31]. The chi-square or the goodness of fit (χ^2^) is usually utilized to access the agreement between the experimental and simulated data, and the lower the χ^2^, the better the agreement and the more reliable the obtained parameters from the fitting. In general, a χ^2^ value of 10−3 and lower is accredited to a better quality of the EIS fitting [32]. In this study, the goodness of fit (χ^2^) values were found to be about 10^−4^, which signifies an appropriate equivalent circuit choice. The constant phase element (CPE), which has been widely used to model surfaces with oxide films and the numerical relationship is well documented [28,33,34], was employed to account for the deviation from ideal capacitive behavior caused by the heterogeneous nature of the inherent surface oxide film formed on stainless steel when exposed aqueous environment [19]. The electrical impedance of a CPE can be calculated according to Equation (3) [19,28].

(3) $$Z_{CPE}=\frac{1}{Q{\left(\omega i\right)}^{n}}$$

where *Q* is a frequency-dependent real constant (Ω^−1^ s^α^), *n* is the quantity that represents the deviation from ideal capacitive behavior, ω is the angular frequency ($\omega =2\pi f\;rads^{-1}$). When *n* approaches unity, an ideal capacitive behavior is observed, and *Q* is equivalent to the film capacitance. The EIS parameters obtained from the fitting are presented in Table 4 and Table 5 for the effect of solutionizing duration and temperature, respectively.

The parameters showing the effect of solutionizing duration (in Table 7) illustrate that both R_ct_ and Rf increased for the solutionized samples compared with the untreated A29 alloy. Quantitatively, the R_ct_ and R_f_ for the sample T30 (solutionized for 30 min) were increased by 68 and 47%, respectively. In contrast, for sample T60, they were increased by 88 and 59%, respectively, and by 126 and 66%, respectively, for sample T120. It is observed from this analysis that the higher resistance of the solutionized samples results from both the charge transfer resistance and the resistance of the surface oxide film and that the oxide film resistance also increases with solutionizing duration. The total impedance (R_T_), which is the sum of the R_ct_ and R_f,_ was used to estimate the protective performance (ε_eis_), which shows the increasing protectiveness of 46 to 60% with increasing solutionizing duration from 30 to 120 min. Similarly, for the effect of solutionizing temperature on the electrochemical behavior of UN08029 alloy (as shown in Table 8), the R_ct_ increases with increasing the solutionizing temperature until 1200 °C, which then drops. However, the R_f_ values remain almost unchanged with increasing solutionizing temperature. Thus, the R_T_ shows 45, 52, 60, and 26% improvement in the corrosion protection performance for sample solutionized at temperatures of 1000, 1100, 1200, and 1300 °C, respectively. These EIS results obtained substantiated the LPR and CPDP measurements.

#### 3.2.5. Corroded Surface Characterization

**Figure 12:**
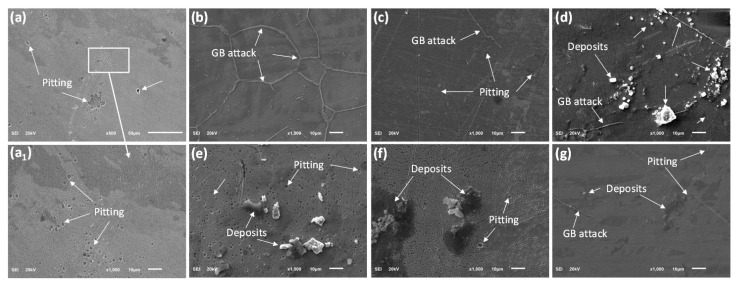
SEM analysis of the exposed area to corrosion in 3.5% NaCl solution after 1 h for samples (**a**,**a_1_**) A29, (**b**) T30, (**c**) T60, (**d**) T120 (or AT12), (**e**) AT10, (**f**) AT11 and (**g**) AT13.

**Figure 13:**
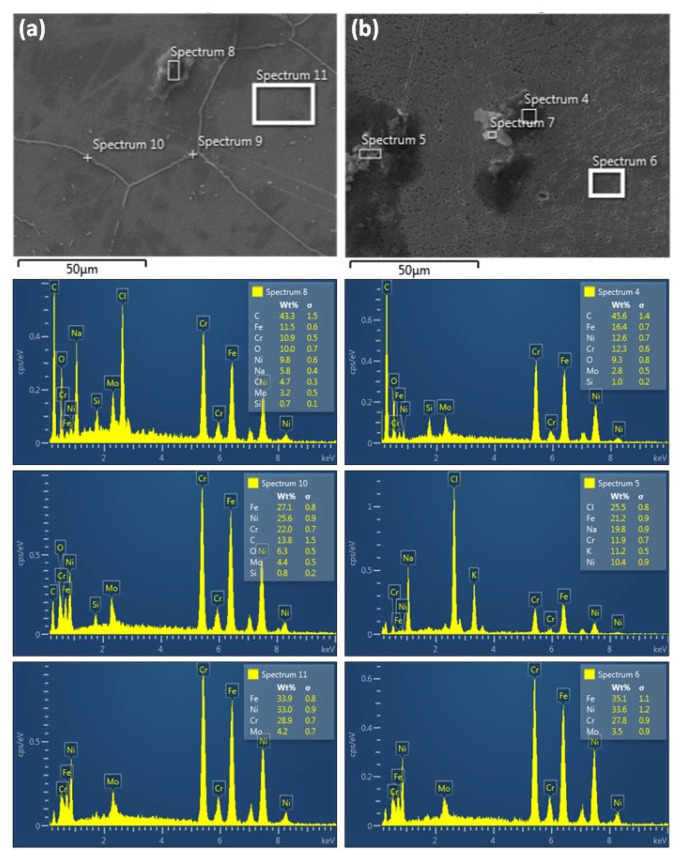
Typical EDS analysis of the corroded surface for samples (**a**) AT11 and (**b**) T30.

The SEM and EDS analyses of the corroded surfaces are presented in Figure 12 and Figure 13, respectively. Several pits at the microscale can be observed on the exposed surface of the as-received sample A29 (Figure 12(a,a_1_)). These pits range between 1–3 µm in diameter and vary in depth but are mostly shallow. In comparison, on the corroded surfaces of the solutionized samples at different duration shown in Figure 12b–d, lesser or no pitting was observed at the same scale. However, for sample T30, a preferential grain boundary etching effect (GB attack) and subsequent deposition on the grain boundary were observed. The EDS analysis of the deposits on the grain boundary, as shown in Spectrum 10 of Figure 13a, shows that the deposits are corrosion products consisting mostly of oxides and carbides of the Fe, Ni, and Cr. Similar deposits are observed within the grains at the initial stage; hence, they can only be well observed at much higher magnification. Spectrum 8 of Figure 13a also shows the composition of typical deposits within the grains compared with the matrix composition shown in spectrum 11. The GB attack or etching effect was also observed in samples T60 and T120. However, the severity reduces with duration showing that these samples exhibited better resistance in comparison. Furthermore, no apparent pitting was observed on the corroded surface of sample T120 (Figure 12d).

Similarly, Figure 12e–g shows the representative corroded surfaces for the samples solutionized at different temperatures, and it is observed that the density of the micro pitting increases for samples AT10 and AT11 in comparison with that of sample A29. Though the density of pits was higher in these samples, the pits were much shallow and spread mostly on the surface. Both GB attack, micro pits as well as corrosion deposits can be observed on the surface of sample AT13. The sample actively corroded uniformly with the presence of micro pitting, which causes the deterioration of its corrosion resistance. Figure 13b shows the EDS analysis of the deposits (Spectrum 4), which are similar in composition to those observed in the grain boundaries, only that the carbon content is higher while Fe, Ni, and Cr are lower. This indicates that the sample actively corrodes uniformly. The content of the Fe, Ni, and Cr in the grain boundaries is associated with increased reactivity due to the availability of dangling or unbonded atoms. Contrastingly, sample AT12 exhibited the least surface damage, as evident from Figure 12d.

## 4. Conclusions

The effect of solutionizing duration and temperature on the electrochemical corrosion and pitting resistance in 3.5% NaCl solution of cold rolled super austenitic stainless UN08029 alloy were investigated in this study. The samples were solutionized at a constant temperature for a duration of 30, 60, and 120 min, and a temperature range of 1000–1300 °C for a fixed duration was considered during the solution annealing treatment. The following are some conclusions from the study:

1. The elongated columnar grains recrystallized to uniform equiaxed grains during the solution annealing. Increasing the solutionizing duration and temperatures resulted in grain growth. This was associated with increased grain boundary migration due to increased thermal kinetics at longer duration and higher temperatures.
2. The polarization resistance measurement showed that the corrosion resistance of the untreated alloy sample was increased by about 67, 68, and 76% after solutionizing for a duration of 30, 60, and 120 min, respectively. Similarly, the resistance of the alloy was also increased by about 72, 74, 76, and 42% after solutionizing at temperatures of 1000, 1100, 1200, and 1300 °C, respectively.
3. Though the pitting potential for the untreated alloy sample is higher than that of the solutionized samples, the hysteresis loop for all solutionized samples was much lower in comparison. Based on the pitting potential and the hysteresis loop, which defined the pitting and repassivation properties of the alloys, the sample solutionized at 1200 °C for 120 min (sample T120 or AT12) showed an optimum combination of pitting resistance parameters, thus demonstrating the least pitting corrosion damage.
4. The total charge transfer resistance obtained from the electrochemical impedance measurement shows an increasing protectiveness of 46 to 60% with increasing solutionizing duration from 30 to 120 min. In addition, a 45, 52, 60, and 26% improvement in the corrosion protection performance for sample solutionized at temperatures of 1000, 1100, 1200, and 1300 °C, respectively.
5. The superior corrosion and pitting resistances for the sample solutionized at 1200 °C for 120 min (sample T120 or AT12) were attributable to larger grain size and spontaneous formation of the dense and compact passive film, which conferred it with a higher charge transfer resistance and faster repassivation property.

## Figures and Tables

**Table 1:** Elemental composition of the UNS N08029 alloy (in wt.%).

Cr	Ni	Mo	Cu	Mn	Si	N	C	P	Fe
26.7	32.8	4.4	0.9	<2.0	<0.7	<0.1	<0.02	<0.02	32.45

**Table 2:** The elemental composition from EDS analysis of the phases identified in the images in Figure 4.

Phase	Fe	Cr	Mo	Ni
γ	36.1	26.7	4.4	32.8
σ	31.4	31.6	6.2	30.8
Carbide (M_23_C_6_)	30.2	36.3	13.6	20

**Table 3:** LPR parameters for the samples solutionized for different durations.

Sample ID	R_p_ (kΩ·cm^2^)	I_corr_ (µA/cm^2^)	ε_lpr_ (%)
A29	101.8	0.260	-
T30	311.8	0.084	67.4
T60	314.8	0.083	67.7
T120	421.4	0.062	75.8

**Table 4:** LPR parameters for the samples solutionized at different temperatures.

Sample ID	R_p_ (kΩ·cm^2^)	I_corr_ (µA/cm^2^)	ε_lpr_ (%)
A29	101.80	0.260	-
AT10	360.80	0.072	71.8
AT11	388.20	0.067	73.8
AT12	421.40	0.062	75.8
AT13	174.40	0.149	41.7

**Table 5:** The Tafel extrapolated data and pitting parameters obtained from the CPDP measurement for the samples heat-treated at different solutionizing durations.

Sample ID	β_a_ (mV/dec)	β_c_ (mV/dec)	I_corr_ (µA/cm^2^)	E_corr_ (mV)	CR (mpy)	ε_cp_ (%)	E_pit_ (mV)	I_p_ (µA/cm^2^)	E_p_ (mV)	E_pit_ – E_p_ (mV)
A29	543.9	270.8	0.94	−144	0.151	-	965.0	23.66	747.2	217.8
T30	281.4	145.7	0.21	−103	0.033	77.7	859.9	33.10	823.5	36.4
T60	225.4	114.9	0.15	−162	0.024	84.0	944.7	26.47	819.9	124.8
T120	225.4	114.9	0.12	−83.3	0.020	87.2	917.4	32.32	831.1	86.3

**Table 6:** Tafel extrapolation and pitting parameters obtained from the CPDP measurement for the samples heat-treated at different solutionizing temperatures.

Sample ID	β_a_ mV/dec	β_c_ mV/dec	I_corr_ (µA/cm^2^)	E_corr_ (mV)	CR (mpy)	ε_cp_ (%)	E_pit_ (mV)	I_p_ (µA/cm^2^)	E_p_ (mV)	E_pit_ – E_p_ (mV)
A29	543.9	270.8	0.940	−144	0.151	-	965.0	23.66	747.2	217.8
AT10	256.4	106.9	0.143	−87.8	0.023	84.8	883.3	32.74	815.0	68.3
AT11	310.8	107.9	0.137	−123	0.022	85.4	875.4	53.52	830.5	44.9
AT12	225.4	114.9	0.123	−83.3	0.020	86.9	917.4	32.32	831.1	86.3
AT13	299.1	128.7	0.425	−133	0.068	54.8	880.8	34.29	811.8	69.0

**Table 7:** EIS parameters obtained from the EC fitting for the samples solutionized for different durations.

Sample ID	R_s_ Ω·cm^2^	R_ct_ kΩ·cm^2^	CPE_dl_ µF/cm^2^	n_dl_	R_f_ kΩ·cm^2^	CPE_f_ µF/cm^2^	n_f_	R_T_ (R_ct_ + R_f_) kΩ·cm^2^	ꭓ^2^ ×10^−4^	ε_eis_ (%)
A29	8.24	90.56	1.01	0.92	0.32	61.19	0.81	91	1.3	-
T30	8.29	152.40	8.52	0.56	15.30	80.14	0.92	168	9.6	45.81
T60	8.07	170.70	5.77	0.52	19.20	112.00	0.91	190	16.9	52.14
T120	7.74	204.50	0.18	0.97	21.38	95.81	0.91	226	3.8	59.77

**Table 8:** EIS parameters obtained from the EC fitting for the samples solutionized at different temperatures.

Sample ID	R_s_ Ω·cm^2^	R_ct_ kΩ·cm^2^	CPE_dl_ µF/cm^2^	n_dl_	R_f_ kΩ·cm^2^	CPE_f_ µF/cm^2^	n_f_	R_T_ (R_ct_ + R_f_) kΩ·cm^2^	X^2^ × 10^−4^	ε_eis_ (%)
A29	8.24	90.56	1.01	0.92	0.32	61.19	0.81	91	1.3	-
AT10	7.82	141.70	24.58	0.50	23.73	84.71	0.88	165	7.2	45.06
AT11	8.03	164.00	67.25	0.79	24.09	80.50	0.91	188	14.0	51.68
AT12	7.74	204.50	0.18	0.97	21.38	95.81	0.91	226	3.8	59.77
AT13	7.90	94.20	206.5	0.90	28.96	101.4	0.93	123	4.6	26.21

## Data Availability

Not applicable.
